# Infection dynamics and persistence of hepatitis E virus on pig farms – a review

**DOI:** 10.1186/s40813-021-00189-z

**Published:** 2021-02-05

**Authors:** M. Meester, T. J. Tobias, M. Bouwknegt, N. E. Kusters, J. A. Stegeman, W. H. M. van der Poel

**Affiliations:** 1 Farm Animal Health unit, Department of Population Health Sciences, Faculty of Veterinary Medicine, Utrecht University, Utrecht, the Netherlands; 2 Vion Food Group, Boxtel, the Netherlands; 3 Wageningen Bioveterinary Research, Lelystad, the Netherlands

**Keywords:** HEV, Transmission, Compartmental model, Risk factors, Zoonosis, Veterinary public health, On-farm persistence, Environmental contamination, Risk mitigation

## Abstract

**Background:**

Hepatitis E virus (HEV) genotype 3 and 4 is a zoonosis that causes hepatitis in humans. Humans can become infected by consumption of pork or contact with pigs. Pigs are the main reservoir of the virus worldwide and the virus is present on most pig farms.

**Main body:**

Though HEV is present on most farms, the proportion of infected pigs at slaughter and thus the level of exposure to consumers differs between farms and countries. Understanding the cause of that difference is necessary to install effective measures to lower HEV in pigs at slaughter. Here, HEV studies are reviewed that include infection dynamics of HEV in pigs and on farms, risk factors for HEV farm prevalence, and that describe mechanisms and sources that could generate persistence on farms. Most pigs become infected after maternal immunity has waned, at the end of the nursing or beginning of the fattening phase. Risk factors increasing the likelihood of a high farm prevalence or proportion of actively infected slaughter pigs comprise of factors such as farm demographics, internal and external biosecurity and immunomodulating coinfections. On-farm persistence of HEV is plausible, because of a high transmission rate and a constant influx of susceptible pigs. Environmental sources of HEV that enhance persistence are contaminated manure storages, water and fomites.

**Conclusion:**

As HEV is persistently present on most pig farms, current risk mitigation should focus on lowering transmission within farms, especially between farm compartments. Yet, one should be aware of the paradox of increasing the proportion of actively infected pigs at slaughter by reducing transmission insufficiently. Vaccination of pigs may aid HEV control in the future.

## Background

Hepatitis E virus (HEV) is the main cause of viral hepatitis in humans worldwide. Human infections with HEV are often asymptomatic, but can cause acute liver failure or chronic infections leading to liver fibrosis and cirrhosis and neurological illnesses [1]. There are at least eight different HEV genotypes (gt) of which five (gt 1 (HEV-1), 2, 3, 4 & 7) are found to infect humans [1]. Clinical cases of HEV in industrialized countries are increasingly caused by the zoonotic gt’s HEV-3 and HEV-4, with domestic pigs as main reservoir [2].

HEV infections in pigs are thought to run a subclinical course, but at post-mortem examination a multifocal lymphoplasmacytic hepatitis and focal hepatocellular necrosis can be observed microscopically [3]. Salines et al. reviewed 45 swine seroprevalence studies and found seroprevalences on country level ranging from 8 to 93% [4]. Reported farm-level seroprevalence (i.e. the percentage of farms with at least one seropositive animal) was higher with fourteen out of fifteen studies reporting a farm-level seroprevalence ranging between 60 and 100% [4]. These data suggest that HEV is likely present, or has been present, on nearly every commercial pig farm worldwide.

Strains circulating in pigs and humans have a high sequence similarity, suggesting that transmission of HEV between pigs and humans is common [5, 6]. Pig-to-human transmission of HEV can occur via the consumption of inadequately cooked pork, and liver in particular [7]. The human risk of foodborne HEV depends on the infection status of slaughter pigs. In case pigs at slaughter have an active HEV infection, meaning that they are viremic or HEV is present in feces or liver, the probability for pork consumers to be exposed to HEV is high [8]. However, large differences in the proportion of pigs slaughtered with an active infection are observed between countries and between farms within countries [4]. Understanding the causes of these differences is key to lowering the exposure of humans to HEV.

To identify factors determining the proportion of pigs delivered to slaughter with an active HEV infection and to install effective measures to decrease this proportion, it is important to understand the infection dynamics of HEV. Therefore, the scope of this review entails 1) HEV infection dynamics in individual pigs and on pig farms, 2) risk factors for within-farm transmission of the virus, 3) mechanisms of persistence of HEV in pig farms, and 4) current knowledge on and suggestions for mitigation of the risk of HEV infections for humans.

## HEV infection dynamics

### HEV infection dynamics in individual pigs

**Fig. 1:**
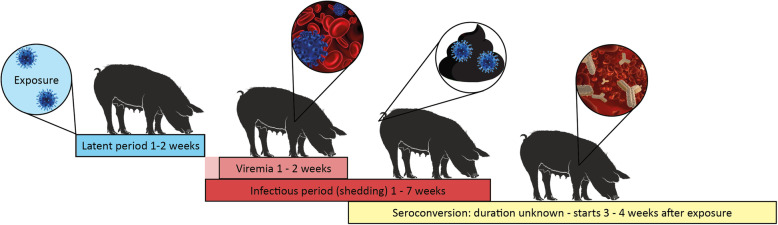
Course of HEV infection as observed in experimentally infected pigs

To understand HEV infection dynamics on farms, knowledge about the infection dynamics in individual pigs is essential, in particular the susceptibility, the dynamics of virus excretion upon infection and the development of immunity. These characteristics of HEV infections have been predominantly studied under controlled settings (Table 1 and Fig. 1). Here, characteristics of infection dynamics in individual pigs, retrieved from literature, are reviewed and differences in the outcomes between studies are interpreted.

**Table 1:** HEV infection characteristics in pigs in eight inoculation studies

Reference	Additional information about article	Route of infection	HEV inoculation dose	Latent period (days)	Duration of fecal shedding (days)	Period between exposure and seroconversion (days)
**Satou et al.** [9]	Modelled data from Halbur et al. [3]	IV	10^4.5^ PI_50_	< 7 ^a^	> 7; < 27 ^a^	25.0 (95% CI: 20.9–31.3)
**Dähnert et al.** [10]	Experiment to find minimal infectious dose of HEV in pigs	IV	9,4 * 10 ^5^ copies	range: 9–15	3/4 pigs: 7 (+ − 2.7)	17
			9,9 * 10 ^4^ copies	15	1/4 pigs: 9	23
			4,0 * 10 ^3^ copies	range: 17–23	no viral clearance seen ^b^	27
			7,2 * 10 ^3^ copies	range: 17–30	no viral clearance 74–61 days after shedding started ^b^	27
			6,5 * 10 ^2^ copies	range: 27–30	no viral clearance seen, minimally 18 days ^b^	37
			6,5 * 10 ^1^ copies	range: 21–37	3/4 pigs: range: 11–28	27
			6,5 copies	range: 37–62	no viral clearance seen ^b^	58
**Bouwknegt et al.** [11]	Block 1	IV	2*10^4^ PCR d.u.	3 (range: 2–7)	49 (95% CI 17–141) ^d^	NA
		Contact	Unknown	9 ^c^		NA
	Block 2	IV	1*10^4^ PCR d.u.	3 (range: 2–7)	13 (95% CI 11–17) ^d^	NA
		Contact	Unknown	17 ^c^		NA
**Bouwknegt et al.** [12]		IV	1*10^4^ PCR d.u.	3.2 (95% CI 2.0–4.3)	39.9 (95% CI 27.7–52.1)	15.7 ^e^
		Contact	Unknown	7.2 (95% CI 4.8–9.6)	23.3 (95% CI 18.7–27.9)	20.2 ^e^
**Casas et al.** [13]	No info about PRRSV status of pigs	Oral	2*10^5^ GEs	22 / 25 ^f^	NA	57 ^g^
**Andraud et al.** [14]		Oral	1*10^9^ GEs	6.9 (95% cred. int. 5.8–7.9)	9.7 (95% cred. int. 8.2–11.3)	26.3 (95% cred. int. 23.5–29.5) ^h^
		Contact	Unknown	7.1 (95% cred. int. 3.2–12.3)		
**Salines et al.** [15]	PRRSV coinfected. ~ Same study design as Andraud et al. 2013	Oral	1*10^8^ GEs	12.9 (95% cred. int. 12.8–14.4)	48.6 (95% cred. int. 27.9–84.6)	43.1 (95% cred. int.: 35.7–52.2)
		Contact	Unknown	13.4 (95% cred. int. 8.6–17.1)		
**Salines et al.** [16]	One group of HEV only and one of HEV/PCV2 coinfected pigs	Oral (HEV only)	1*10^7^ GEs	12.3 (95% cred. int. 4.4–25.5)	NA	25.6 (95% cred. int.: 19.3–33.8)
		Oral (HEV/PCV2)	1*10^7^ GEs	11.6 (95% cred. int. 2–21.6)	NA	49.4 (95% cred. int.: 40.4–60.4)

Terms for quantifying HEV according to original article were used. In the review one term, genomic copies, is used. ^a^ Halbur et al. [3], Table 2. ^b^ Because of the long duration of latency a lot of pig couldn’t be followed up long enough to find the end of shedding period. ^c^ Data was censored for 20/37 pigs. ^d^ Based on exposure to shedding of the inoculated animals. ^e^ Sum of the latent period and the period between shedding and seroconversion. ^f^ Five animals died within 3 weeks post inoculation, with pyrexia, shivers, difficult breathing (bacterial systemic infections). Only four pigs were successfully infected of which only two shed and two others seroconverted. ^g^ The two seroconverted pigs had maternal antibodies. ^h^ Outcome was published as additional file of Salines et al. [15]

#### Susceptibility (dose-response relationship)

The probability of an individual to become infected by a pathogen is dose dependent and related to route of exposure. For oral ingestion, Bouwknegt et al. estimated the dose at which the probability of infection equals 50% at 1.4 * 10^6^ HEV genomic copies (PI_50_) [25]. Andraud et al. reported similar results after orally inoculating twelve pigs with different doses, ranging from 10^4^ to 10^8^ HEV copies. Two out of three pigs became infected at a dose of 10^6^ HEV copies [14]. Although oral ingestion is presumed the primary route [26], the intravenous (IV) dose could be estimated more precisely than oral and resulted in a probability of infection per infectious HEV particle of 4.8 * 10^− 3^ [25].

#### Dynamics of virus excretion

Pigs infected with HEV do not immediately become infectious, but first enter a latent state (Fig. 1). In experimental studies, the latent period for HEV is defined as the period between inoculation and HEV excretion in feces [12]. The latent period is also related to dose and challenge route and found to be shorter in IV compared to orally infected pigs. The latent period is also shorter in case of a higher inoculation dose (Table 1) [10]. In IV inoculated pigs, the latent period ranges between 2 and 7 days [9, 11, 12], whereas in orally inoculated pigs, the latent period is 6.9 (95% credibility interval (cred. int.) 5.8–7.9) days [10]. A latent period of 12.9 (95% cred. int. 12.8–14.4) days was observed in HEV orally infected pigs, coinfected with Porcine Respiratory and Reproductive Syndrome virus (PRRSV) [15]. However, because the HEV challenge dose in the coinfected group was tenfold lower than in the HEV-only group it is unclear whether the prolonged latent period was the result of the co-infection, the lower dose or both.

For contact-infected pigs the latent period cannot be determined directly, because the moment of infection is unknown. An estimation of the latent period is usually derived by extracting the time of first HEV shedding from the time of probable exposure to HEV excreted by inoculated pigs. Four studies report the latent period to be between 6 and 19 days in HEV-only contact infected animals and 13.4 days in HEV/PRRSV coinfected animals [11, 12, 14, 15]. So, in general the latent period in pigs lasts around 1 to 2 weeks, but much longer periods have been reported (Table 1).

The end of the latent period, and simultaneously the start of infectivity, is indicated by the first moment of fecal shedding and/or viremia. Viremia was estimated to start 12.6 days post exposure (p.e.) (95% confidence interval (CI) 8.3–17.0) and last 10.5 (95% CI 8.1–13.0) days in contact-infected pigs [12]. Shedding of HEV predominantly occurs via feces and the duration of fecal shedding is therefore often referred to as the infectious period, which lasts 7 to 50 days (see Table 1). The length of the period pigs shed HEV in their feces depends on the route of infection, the inoculated dose and whether the pig is coinfected by another pathogen. In case of oral administration or HEV exposure by contact with infected animals (presumed oral infection), the infectious period lasts 9.7 (95% cred. int. 8.2–11.2) days according to Andraud et al. and 23.3 (95% CI 18.7–27.9) days according to Bouwknegt et al. [12, 14]. The immunomodulating virus PRRSV may cause a slower response of the immune system to HEV and as a consequence prolonged HEV fecal shedding [15].

Aside from fecal shedding, urinary shedding of HEV may occur. HEV is found in kidneys in both experimentally [12, 27] and naturally infected pigs, indicating replication of the virus in kidneys [28]. On top of this, HEV has been isolated from urine of infected pigs [12, 29]. According to Bouwknegt et al. HEV shedding in urine can be observed for up to 65 days post inoculation (p.i.) / p.e., for some pigs long after fecal shedding has ended [12]. In monkeys it is possible to intravenously infect one monkey with urine of another HEV (gt 4) infected monkey [30], indicating the viability of urine shed HEV.

The period of viral fecal shedding in pigs is long compared to other porcine viruses, but infectivity should also be assessed by quantitative evaluation of virus shedding in feces and urine. Pigs most often shed around 10^4^ HEV genomic copies per gram of feces (range 10^3^–10^8^) [14–16]. In urine shedding has not been quantified systematically, yet Bouwknegt et al. (2009) reported one urine sample to have the same C_t_-value (C_t_ = 32.5) in Q-PCR as fecal samples in the acute phase of infection, suggesting that the quantity in urine can reach the same levels as in feces [12].

Pigs are no longer considered infectious to other pigs when shedding and viremia have ended. Nevertheless HEV RNA may persist in the liver, bile and other organs of pigs after the end of the infectious period and thereby the pigs may still be infectious to humans (e.g. [12]).

#### Immune response

Intravenously inoculated and contact-infected pigs both seroconvert around 13 days after the first fecal HEV shedding [12] and orally infected pigs were reported to seroconvert on average 26.3 (95% cred. int. 23.5–29.5) days p.i. (Fig. 1) [15]. Humoral immunity and infectivity are not mutually exclusive as pigs with HEV-antibodies may still be viremic (e.g. 40% of seropositive pigs in a Scottish study [31]) or shed HEV (e.g. 16% of seropositive pigs in a Spanish study) [13, 20]. With regard to immunity one should realize that detection of antibodies does not necessarily imply protection, especially in viral infections where cellular immunity may also play a vital role. Few studies about cellular immunity to HEV in swine are available. Experimental HEV infection did not alter cytokine production or frequencies of different types of T-cells, except for an increased frequency of TGF-β regulatory T cells at 8 weeks p.i. and a decreased frequency of TNF-α and IFN-γ CD4^+^CD8^+^ T-cells in pigs 13 weeks p.i [32]. Cellular immunity may be an interesting target for vaccine interventions (see more in part D).

In conclusion the course of HEV infection in individual pigs varies with the route of inoculation, the viral dose and possibly the influence of immunomodulating viruses such as PRRSV. Whether infection results in pigs that are still actively infected at slaughter is greatly dependent on the age at which pigs become infected. The next section will entail HEV infection in populations of pigs.

### HEV infection dynamics in pig populations

**Fig. 2:**
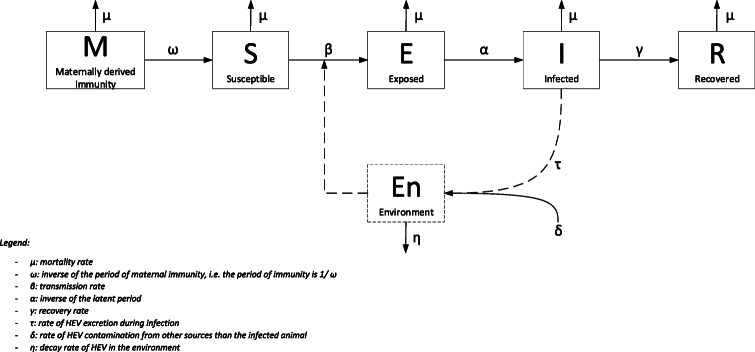
HEV compartmental infection model

The dynamics of HEV infections in individual pigs influence the transmission between pigs, and hence the dynamics in pig populations. The dynamics of infections in populations can be modelled using compartmental models [33]. Dynamics of HEV in populations have been studied using a so-called SEIR (Susceptible – Exposed - Infectious – Recovered) compartmental model and similar models that also account for environmental contamination (En) and waning maternal immunity (M) [34] (Fig. 2).

#### S

In an SEIR-En model at population level, assuming a fully susceptible population, all pigs start in the compartment S. The compartment maternally derived immunity (M) only matters in populations that are not fully susceptible and will be addressed later. The number of susceptible individuals decreases over time, conditional on an increasing number of infected pigs, the number of pigs that one infected pig can infect and the mortality rate (*μ*). Mortality is considered equal for all model compartments as mortality due to HEV infection is negligible in pigs.

#### E

The transition from the susceptible to the exposed compartment is determined by the transmission rate parameter *β*. The total number of pigs in E also depends on the inverse of the latent period (*α*, see section B1 for the latent period of HEV in pigs), as after the latent period they enter the next phase, I. For HEV, *β* is affected by direct (contact within pens) and indirect contact (contaminated pen due to infected pigs) with infectious pigs, as well as by the contaminated environment (via infected pigs in another pen). Three studies quantified the transmission rate parameter for contact and for environmental infections separately [11, 14, 15, 34]. Although direct and environmental transmission are defined differently in those studies and therefore not easily comparable, some estimates are given below. The *β* for direct contact only was estimated at 0.15 per day (95% CI 0.03; 0.31) [14] and for transmission within pens (direct and indirect transmission within pens) at 0.70 (95% CI **1.18 · 10**^**− 3**^; 3.67) [15] and 0.66 (95% CI 0.32; 1.35) [11]. Within-pen environmental transmission (*β* of 2 · 10^− 6^ animals infected per gram/genome equivalents/day **(g/ge/d)** (1 · 10^− 7^; 7 · 10^− 6^)), occurred more than between-pen transmission of HEV (*β* of 7 · 10^− 8^ g/ge/d (5 · 10^− 9^; 3 · 10^− 7^)) [14]. Environmental transmission between pens was found to be a rare event in experimental studies, probably because of strict segregation in pens and avoidance of movement between pens [11, 14].

#### I

The number of infected pigs in time is equal to the number of exposed pigs divided by the duration of latency (see section B1; around 1–2 weeks), minus the number of pigs that recover in that same period of time and the number of pigs that die or go to slaughter.

#### R

Recovery is considered the end of the phase of shedding, thus the rate of recovery per day (*γ*) is 1/17 (average of the infectious period according to oral infection studies (Table 1)). Multiplying *γ* by the number of infected animals per period returns the number of recovered pigs per period. Recovered pigs may become susceptible again, as some report sequentially infected pigs with two different HEV strains throughout their lives (e.g. [18, 20]) and sows with IgG HEV antibodies pre-farrowing that started shedding HEV post-farrowing [20]. However, we expect that return to susceptibility is a minor issue in fattening pigs because they mostly become infected some weeks before slaughter and antibodies will still be protective for infection (see later).

#### En

The dose of infectious HEV in the environmental compartment En primarily results from HEV shedding pigs (*τ*). The quantity of HEV in the environment depends on the number of shedding pigs and the quantity of HEV in feces a pig sheds per day (related to days p.i.) [34]. In addition to shedding pigs, other factors such as contaminated water, feed and rodents may contaminate the environment (*δ*, see also part C persistence). The decay rate of HEV (*η*) corresponds to the proportion of feces and urine passing through slatted floors, the survival of HEV in the environment, and the proportion of HEV eliminated by cleaning and disinfection [34]. Survival of infectious HEV genomic copies outside the pigs body is dependent on time, temperature and interaction with biological and chemical degradants, such as disinfectants, UV-radiation or composting. As far as we know, no studies are available with regards to the survival of HEV in general, or after different cleaning and disinfection routines [35].

In the compartmental model, the basic reproductive number R_0_ indicates the average number of secondary infections produced by one infected individual in a fully susceptible population. One study estimated an R_0_ of 8.8 (95% CI 4–19), based on 2nd and 3rd generation contact infected pigs from one-to-one transmission experiments [11].

However, the use of the SEIR-En model to study infectious disease dynamics assumes random mixing of individuals, whereas in farms pigs are housed in different barns. These barns themselves are further subdivided into compartments and pens that vary in size between farms. The degree at which compartments and barns have contacts that enable HEV transmission depends on various farm management processes as well as internal biosecurity. However, the frequency and intensity of contact between pigs housed in different compartments and barns is considerably lower than that within pens or between adjacent pens [11]. The effect of contact reduction on the transmission of infections can be highly significant as was shown e.g. for Aujeszky’s disease virus (ADV). Even though R_0_ for ADV is higher than 1 in vaccinated finishing pigs indicating sustained transmission in a random mixing population, the combination of vaccination and compartmental housing resulted in eradication of the disease [36]. A better understanding of the transmission of HEV between batches and barns will aid in evaluation of good practices regarding HEV control on farms and design of new interventions. The following sections discuss HEV infection dynamics in farm compartments as well as associations with specific farm management and hygiene practices (risk factors) from an observational epidemiological perspective.

### HEV infection dynamics from birth to slaughter

#### Vertical transmission of HEV

In humans, transplacental infections of HEV (gt 1) occur and can lead to i.e. fetal loss, preterm labor, and hepatic dysfunction in neonates [37–40]. In pigs, the few studies investigating transplacental infection of HEV that are published have contradictory results. Kasorndorkbua et al. studied the effects of HEV in IV infected gestating gilts during late gestation and none of the gilts aborted and fetal viability, birth weight and liver profiles did not differ from piglets of control animals [41]. HEV was neither found in the organs of the fetuses, nor in seemingly healthy born piglets (2003). Hosmillo et al. (2010) detected HEV in the livers of twelve of 59 aborted fetuses of two farms, but the study is inconclusive about whether or not HEV was the cause of the abortions [42]. Nevertheless, the latter study suggests that transplacental infection of HEV in pigs is possible. The incongruence between the two study results could be related to the gestation phase in which sows conceivably were infected.

Although transplacental infections may be possible, it remains unclear if infected sows can give birth to clinically healthy, HEV infected piglets attributing to either E or I. According to multiple studies monitoring pigs from birth to slaughter, pigs are born free from infection [19, 21, 22]. One longitudinal study has found three HEV infected piglets 1 week after birth, but their mothers/nursing sows were PCR negative at that moment so these piglets have probably been infected just after birth instead of as fetus [18]. We conclude that the impact of vertical transmission on within farm HEV dynamics is negligible.

#### Farrowing stage

After birth, suckling piglets acquire maternal immunity against HEV via colostrum from seropositive dams (compartment M, see Fig. 2) [20]. Overall, 50 to 100% of sows have anti-HEV antibodies [43–46]. As a consequence, IgG is demonstrated in 60 to 100% of piglets’ serum in the first weeks of their lives [18, 20, 21, 47], with the highest titers around 1 week of age and decreasing until 9 weeks. Viremia or fecal shedding of HEV is seldomly reported in piglets in the farrowing room (e.g. [18, 24]), presumably because colostral IgG has virus neutralizing capacity [48]. Although HEV shedding sows are observed in the farrowing room [18, 20, 43, 49–52], HEV infections in suckling pigs are seldomly found.

Maternal antibodies can result in a later onset of HEV viremia and seroconversion as demonstrated in the study of Kanai et al., who compared HEV infection in two litters. One litter was delivered by a seropositive dam, the other by a seronegative dam (2010) [19]. Although the infection occurred later in life, a difference in fecal shedding patterns between the two litters was not seen [19]. Krog et al. (2019) tried to repeat the results found by Kanai et al. in multiple litters from one farm and could not discover a difference in the course of infection between litters, yet pigs of sows with high antibody titers did not become shedders as often (73% against 45%, *p* = 0.03) [22] and although less pigs shed virus, equal numbers of pigs seroconverted. Thus, based on this study, acquired maternal immunity does not protect against infection but it may reduce the viral load in feces below detectable levels, lowering the direct transmission and environmental contamination [22]. Contrary, Casas et al. studied the effect of maternal antibody titers on infection in many litters of multiple farms and did not report an effect (see Table 2) [20]. Notwithstanding that effects of maternal immunity on HEV infection are not always observed, in HEV population dynamics models (Fig. 2) maternal immunity has been taken into account, by estimating the probability of infection at 0.08 during 46 days of maternal immunity (41.4; 50.4 days) [34, 48], instead of the baseline of 1 past the period of maternal immunity.

**Table 2:** Infection dynamics of HEV in pig farms, reported in eight longitudinal studies

Reference	Size of study population	Age in weeks when 20% of pigs or less have maternal IgGs	Age of first shedder / viremic pig (weeks)	Age when most pigs shed HEV (weeks)	Cumulative incidence of infection (based on shedding / immune response)	Age range of detection IgG positive pigs (weeks)
**LeBlanc et al.** [17]	1 farm, 51 piglets from 51 litters	No maternal antibodies detected	2	18	98.4%	NA
**De Deus et al.** [18]	1 litter	9	1 (viremia)	12 and 15	NA	15–22
**Kanai et al.** [19]	litter 1 with mat. IgG	7 (based on ELISA index value instead of % of animals)	4	Continuously from 6 to 14	100%	9–17
	litter 2 without mat. IgG	No maternal antibodies detected	4	Continuously from 6 to 13	100%	10–17
**Casas et al.** [20]	Farm 1, 5 sows, 20 piglets	8 (based on sampling only at week 7 and 13, no information about period in between)	6 (estimate based on IgM)	NA	80% (based on IgM, underestimation)	18–25
	Farm 2, 5 sows, 20 piglets	12	12 (based on IgM)	NA	37% (based on IgM)	18 - NA
	Farm 3, 5 sows, 20 piglets	6	6 (based on IgM)	NA	90% (based on IgM)	13–18
	Farm 4, 5 sows, 20 piglets	12	3	NA	53% (based on IgM)	13 - NA
	Farm 5, 5 sows, 20 piglets	13	3	NA	90% (based on IgM)	13 - NA
	Farm 6, 5 sows, 20 piglets	10	3	NA	100% (based on IgM)	13 - NA
**Feng et al.** [21]	1 farm, 32 pigs from unknown sows	8	Between 4 and 9 (viremia)	NA	100% (based on seroconversion of all animals)	11–17
**Krog et al.** [22]	group 1 and 2: low/intermediate mat. IgG	No maternal antibodies detected	13	15	73%	11–13
	group 3 high mat. IgG	7 (based on normalized OD values instead of % of pigs with IgGs)	13	15	45%	11–13
**Motoya et al.** [23]	1 farm, 7 piglets from one litter	Study started at 6 weeks of age, no maternal antibodies detected	9	11–14 all shed	100%	11–15
**Salines et al.** [24]	Farm 1, 3 batches, 120 pigs in total	6 (based on average optical density)	14–18	19	41%	18/22 - NA
	Farm 2, 3 batches, 120 pigs in total	6 (based on average optical density)	1 week in 1 batch; 6–10 weeks in 2 batches	7	85%	10/14 - NA
	Farm 3, 3 batches, 120 pigs in total	6 (based on average optical density)	1 week in 1 batch; 10–14 weeks in 2 batches	14	80%	14/18 - NA

#### Nursery stage

On farms, the first shedder of HEV is usually detected in the nursery room (Table 2) whereas some studies find zero shedding and/or viremic weaned pigs [20, 22]. Casas et al. for example has followed up HEV dynamics in six farrow-to-finish farms and reported a similar infection pattern in five farms, but in one farm the first shedder was detected 6 to 9 weeks later than on the other farms (post nursing phase) [20]. Salines et al. also found one farm with a noticeable earlier average age at shedding (~ 6 weeks) than on the other farms [24]. None of the studies report a peak in the number of shedders in the nursing phase. In the MSEIR-En model, specific for nursery compartments, most pigs will be in M and S, some in E and even less in I. None of the pigs will be recovered. In the best case the environment will hardly be contaminated because the pigs are not yet shedding. Still, cleaning and disinfection procedures on the farm may determine the importance of environmental contamination for the next batch of pigs.

#### Fattening stage

After moving the pigs to the fattening room a steep increase is seen in the number of HEV shedders on farms (see Table 2). In a meta-regression study where HEV prevalence in feces was constructed according to age, the maximum prevalence was predicted at 11 weeks of age, in the beginning of the fattening period [4], so the compartmental model for fatteners may temporarily contain the highest number of pigs in I. Moreover, quantitative analyses of fecal shedding showed that level of shedding for most pigs is highest between 12 and 15 weeks of age [22, 23] (En affects I the most in these weeks). Viremia occurs between 11 and 18 weeks of age, and coincides with increasing serum IgM levels [17, 18, 21]. IgG is detected from the end of viremia and the number of seroconverted pigs continues to rise until they reach slaughter age (≥25 weeks) [17, 20, 22, 53]. Therefore, in slaughter pigs, seroprevalences up to 100% are reported, (proportion of recovery nearly 1) as mentioned earlier.

A longer shedding period or a lower transmission rate, resulting in fewer recovered slaughter pigs, will result in a higher probability of finding actively infected pigs being slaughtered. Therefore identification of factors that affect the infection dynamics in farms is needed.

### Factors causing differences in infection dynamics on farms

**Fig. 3:**
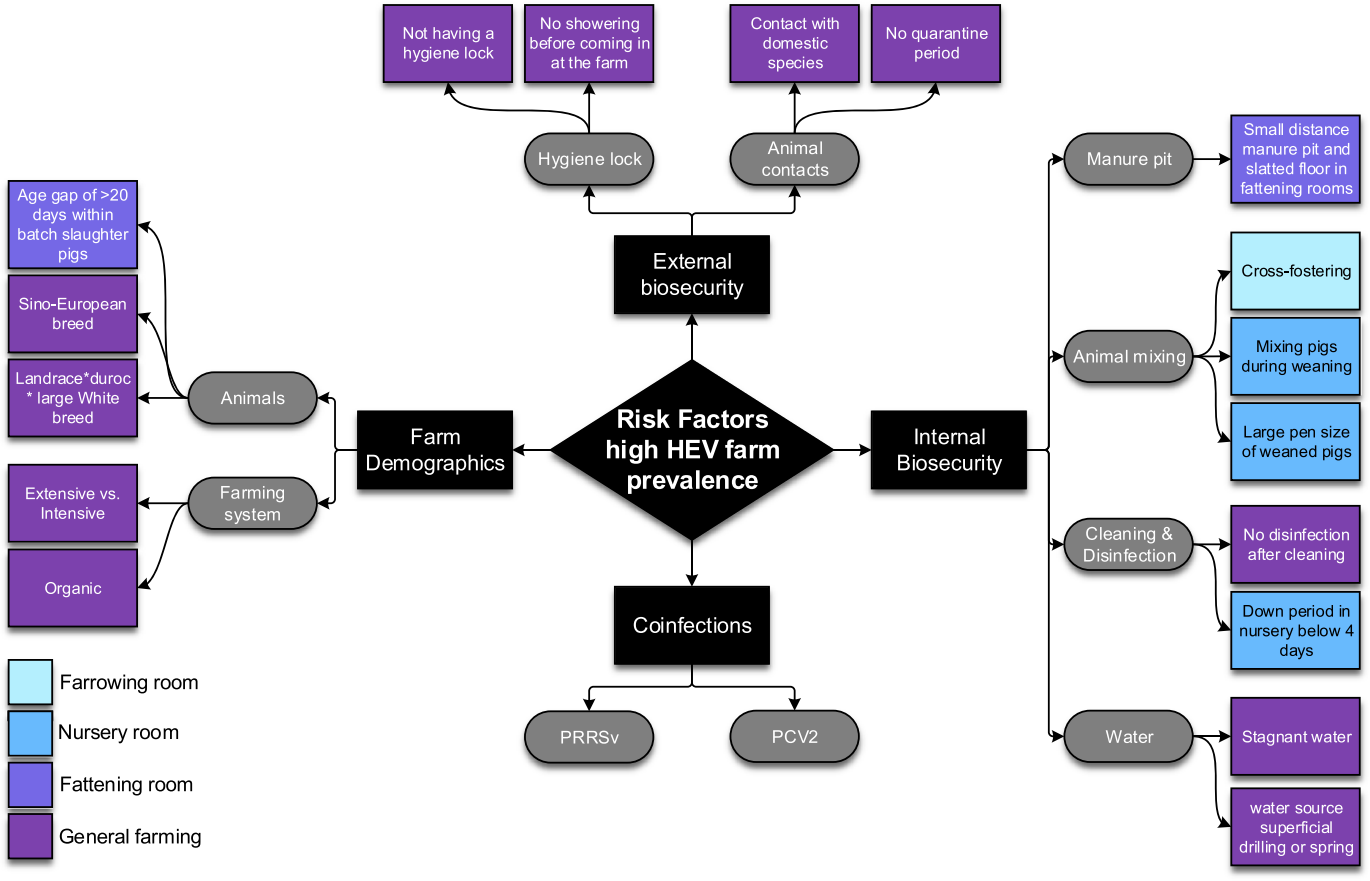
Flowchart of the significant risk factors for a high farm level HEV prevalence or seroprevalence. The black and grey shapes contain names that categorize the risk factors. The different colors of blue and purple represent farm compartments. The given risk factors represent a high HEV prevalence or seroprevalence

Observed differences between infection dynamics of HEV on farms can be explained by plausible causal mechanisms and stochasticity as well as potential risk factors and confounders. In literature risk factors are predominantly described from studies that seek for associations between HEV (sero) prevalence at slaughter and farm management, likely interacting on the transmission rate and environmental compartment from the MSEIR-En model. The risk factors and their assumed consequence on HEV infection dynamics are discussed and depicted per category in Fig. 3.

#### Farm demographics

Various farm production systems are associated with HEV prevalence [54–56]. The factor ‘farming system’ comprises management measures that probably have a direct effect on aspects of the MSEIR-En modelling for HEV transmission. For instance, extensively farmed pigs have more feco-oral contact due to fewer slatted floor surface, more possibilities for rooting behavior and in addition may come into contact with other animal species. Extensively farmed pigs may therefore have more, or a different source of exposure to HEV than intensively reared pigs. In fact organically raised pigs were reported to have a higher within-farm seroprevalence than pigs raised conventionally [56]. Lopez-Lopez et al. (2018) reported the odds of a high HEV seroprevalence significantly higher for extensively producing farms than for intensively producing farms [54]. On the contrary, De Oliveira-Filho et al. found no difference between intensive (indoor) farms and extensive (free-ranging) farms (2017). Semi-intensive production was associated with a lower HEV seroprevalence, although the definition of semi-intensive was not given [55].

Shipment of younger pigs to slaughter may increase the likelihood of slaughtering actively infected pigs, because pigs are often infected early in the fattening period. Walachowski et al. have found that a difference of more than 20 days in age within the same slaughter batch versus a difference of less than 20 days has a relative risk of 6.0 (1.3–66.0) for having RNA positive livers in slaughter pigs of that farm [57]. A batch with a large variation in age may indicate that the batch contains a higher proportion of younger pigs that reached the ideal slaughterweight earlier, and thus a higher proportion of pigs may also be actively infected at slaughter.

The other farm demographics risk factor that has been associated with HEV prevalence is pig breed. Walachowski et al. reported that pigs from specific breeds (Landrace*duroc*large White and Sino-European) had RNA positive livers at slaughter more often than the Landrace*Large White breed [57]. This may have to do with susceptibility of the breed but could also be confounded by the type of farms that keep those breeds or the growth efficiency of different breeds and thus the age at which pigs are slaughtered. Noteworthy, no information is available on association the between risk of HEV infection and pig sex, castrated or intact boars or dam parity.

#### External biosecurity

External biosecurity encompasses all measures that prevent entry of pathogens into a herd [58]. Regarding HEV, this would mainly imply prevention of introduction of contaminated feces or materials and infected animals, porcine as well as non-porcine. Not having a hygiene lock is reported to correlate with higher HEV seroprevalence on pig farms [54]. Furthermore, requiring showering and providing boots before entrance of the farm are protective factors for HEV introduction [59]. Boots seem especially protective when they are exclusively used for swine production [57]. Not having, or not using a hygiene lock can lead to introduction of HEV in the farm (compartments). If pigs in those compartments have not been exposed to HEV before that, and are close to slaughter age, this can cause a large number of actively infected pigs at slaughter.

HEV can also be introduced via contact of the farmed pigs with other animals. Lopez-Lopez et al. discovered that contact of pigs with domestic species like cats and dogs increases the odds of having viremic sows and fattening pigs on the farm [54]. However, this finding was not confirmed by others [57, 60]. Although various studies have reported HEV seroprevalences of around 10 to 30% in domestic carnivores, not a single study has reported HEV-shedding or viremia among these animal species [61]. Thus, it can be questioned whether seropositive carnivores transmit the virus to pigs, or vice versa and probably the latter is more likely (e.g. by feeding them pork). The animals could serve as vector on the farm, spreading HEV contaminated fecal material. Wildlife like wild boar and deer have been demonstrated to be susceptible to HEV and could interact with and transmit HEV to pigs, in particular when kept outside [62]. Interaction with wildlife however has not come forward as risk factor for HEV on pig farms.

The introduction of new, HEV infected, pigs to the herd, for example gilts, can be a source for introduction of the virus. Applying a quarantine period could reduce the risk of introduction and is also associated with a lower HEV seroprevalence [54]. Moreover, acclimatizing gilts, by feeding them feces and placentas of sows, was negatively associated with the proportion of positive livers at slaughter on farm level [57]. Gilt acclimatization has the opposite effect of quarantining the gilts, because it intends to infect gilts with farm related micro-organisms, instead of awaiting recovery from potential infections to the farm. The negative association between HEV seroprevalence and acclimatization could indicate that replacement gilts are more often susceptible for HEV than sows, which would correspond with the course of infection and age dependent seroprevalence of HEV (e.g. [43]).

#### Internal biosecurity

Internal biosecurity concerns measures that reduce spread of pathogens within a farm. Within a farm HEV can spread i) between animals within the same pen, ii) between animals in different compartments or iii) via environmental contamination.

Transmission within pens is inevitable because of exposure to infectious excreta and secretions of pen mates. HEV could be distributed over multiple pens and compartments by a manure pit that is connected between compartments. A short distance between the manure in the pit and the slatted floor in fattening rooms is recognized as a risk factor for a higher HEV seropositivity [57]. A shorter distance is equivalent to a greater chance of exposure to the manure of all other pigs of the same and perhaps of other compartments.

Several mixing practices have also been reported to increase the risk of a high HEV prevalence, namely cross-fostering, regrouping piglets during weaning and (indirectly resulting in mingling of litters) a pen size of more than 26 pigs in nursery [57]. How mixing influences the farm infection dynamics can be seen in the longitudinal HEV studies described earlier. For example, Nakai et al. (2006) compared three farms; the farm where litters were not mingled during weaning had the lowest HEV prevalence [63]. LeBlanc et al. (2007) found HEV infection in young piglets on a farm that received weaned pigs at 2 weeks of age from different suppliers (inevitably meaning a lot of mixing) [17]. As can be seen from these examples, mixing pigs accelerates the transmission and consequently seroconversion occurs at a younger age. On the one hand, acceleration of the transmission can cause a higher incidence of HEV on a farm, thus elevating the proportion of active infections at slaughter. On the other hand, because the infection manifests at a younger age, it can also imply that all pigs may already be recovered when ready for slaughter. This paradox will also be covered in part D.

The importance of the environmental contamination for HEV transmission within herds is evidenced in field studies. Not disinfecting barn areas after cleaning [55] and having a down period of less than 4 days in nursery compartments are associated with a high HEV prevalence [57].

Contaminated water can also attribute to the environmental exposure of HEV. Twice, water has been recognized as a risk factor for higher HEV prevalence. Firstly, when using a mixed drinking water system with partly stagnant water [55] and secondly, when a spring or a drill of less than fifty meters deep is used as drinking water source [57]. Both risk factors are related to a higher probability that the water becomes contaminated by HEV. The abovementioned risk factors suggest that adequate cleaning and disinfection and a long down period are crucial to reduce the contribution of the environmental attribution to spread of HEV within farms.

#### Coinfections

Coinfections refer to a situation in which two or more species of pathogens coexist in the same host [64]. Case reports have shown coinfections in pigs of HEV and PRRSV, and HEV and PCV2 [65–67]. PRRSV and PCV2 are immunomodulating pathogens, although the specific impacts of these viruses on the immune system have not yet been fully uncovered [68, 69]. The effects of PRRSV and PCV2 on HEV infections in pigs have been studied experimentally, as mentioned in part B earlier and longitudinally on three pig farms [24]. In the longitudinal study a PRRSV or PCV2/PRRSV infection, before or during a HEV infection was associated with a higher age at HEV shedding and a higher age at HEV seroconversion [24]. A PCV2/PRRSV pre- or coinfection was additionally associated with a longer fecal shedding period (meaning a lower *γ*) and resulted in more HEV infected livers at slaughter [24]. A drawback of this study is that all HEV-only infected pigs came from a single farm and on that same farm the majority of PCV2 and PRRSV infections occurred after the HEV infections. Consequently, disentangling pre- and coinfections from the farm effect is a challenge in that study.

Even if PCV2 and PRRSV contribute to an increase of the number of actively infected slaughter pigs, it is hard to act upon it, considering PRRSV and PCV2 are two of the most herd persistent pig viruses and difficult to eliminate from farms. In the longitudinal study that looked at coinfections for instance, 53.7% of the pigs experienced all three infections and only 8.6% got only infected by HEV [24]. In future strategies to lower HEV in pig farms, the persistency of PRRSV and PCV2 may have to be taken into account more specifically.

To summarize, a complex interplay between farm demographics, external and internal biosecurity and the presence of immunomodulating coinfections can influence HEV infection dynamics on farms. The exact mechanisms behind most risk factors remain to be uncovered. The relative importance of the different factors is contingent on HEV reservoirs on farms and whether and where HEV persists on pig farms.

## Persistence of HEV on farms

### Within-farm persistence or reintroduction?

After introduction of a pathogen, infections either fade-out, or infections persist on farms. Based on the observed high farm-level seroprevalences (i.e. [56, 70, 71]) and the high R_0_ [9, 11], HEV persistence on farms seems conceivable. Although frequent (re-)introduction of the pathogen may also contribute to high farm-level seroprevalence this seems unlikely. Phylogenetic analyses of HEV strains indicate on-farm persistence for two reasons. Firstly, farms often have a unique HEV strain, which can be seen in phylogenetic trees as a separate cluster for every farm [6, 72, 73]. Secondly, even multiple strains sampled from the same farms, with a long sampling interval, are often closely related [6, 72]. A French study did not find this close relationship between strains from a farm sampled twice, but multiple introductions on that farm could have occurred due to buying grower pigs from multiple origins [74].

Altogether, we conclude that HEV can persist on pig farms at least for several years. Persistence is only feasible if R_0_ > 1, allowing major outbreaks to occur when sufficient susceptible animals are added to the population to spur the chain of infection and contacts between batches and with contaminated environments are efficient. Mechanisms for the persistence on pig farms are presented and weighted below.

### Mechanisms of HEV persistence within farms

#### Persistence in animal populations

Although optimal pig farming is organized strictly on batch level, day-to-day practice often compromises this principle and thereby enables transmission of pathogens between batches. This process corroborates with findings that HEV shedding is associated with movement of pigs and that mixing pigs is a risk factor for a high HEV prevalence [57].

### Environmental persistence

**Fig. 4:**
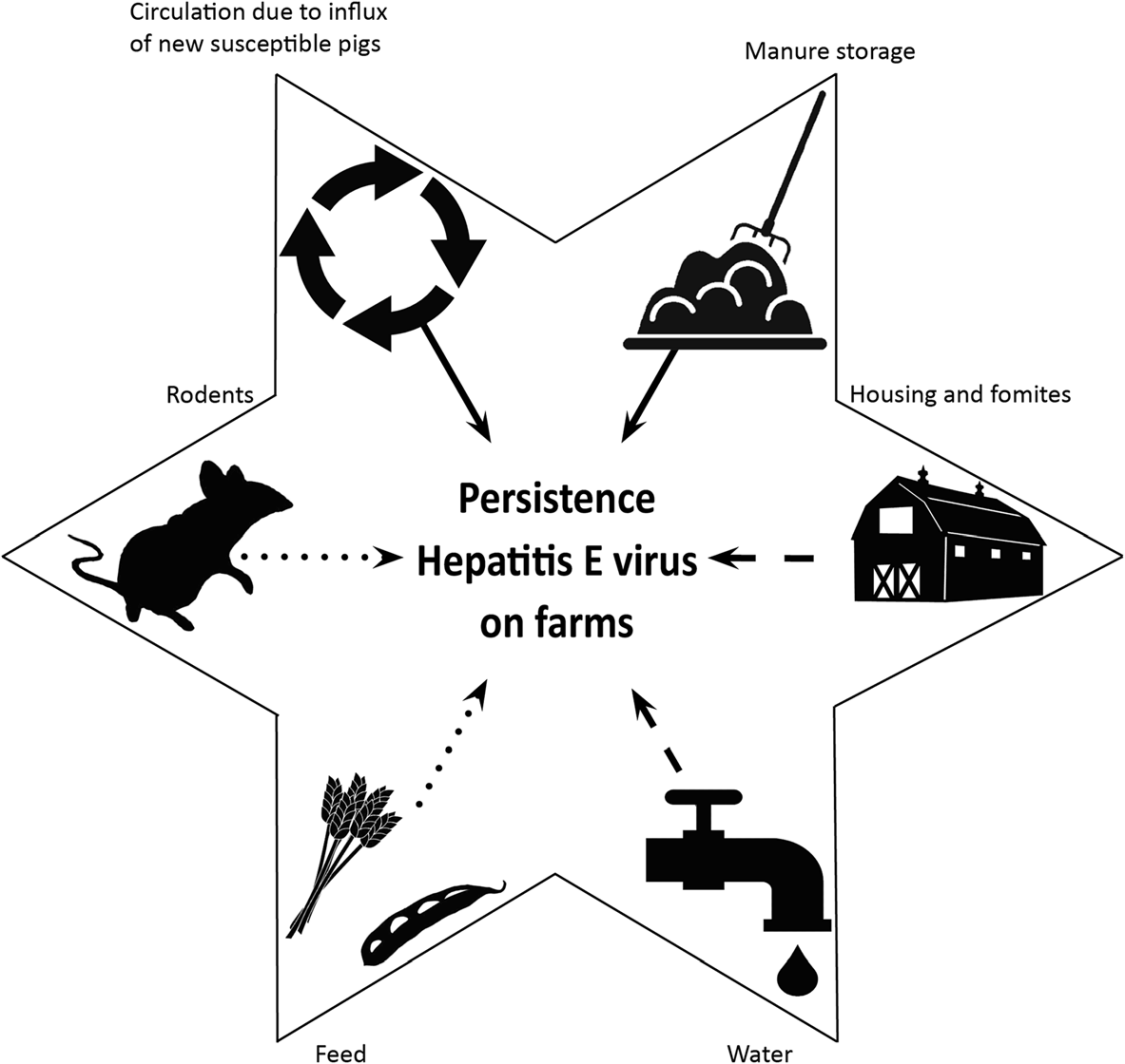
Potential mechanisms for HEV persistence on pig farms. Solid arrows represent mechanisms of persistence confirmed by literature, dashed arrows may be sources of persistence, yet more research is needed to conclude on these sources and dotted arrows are unlikely sources of persistence

As HEV excretion predominantly occurs via feces (and possibly urine), the manure storage, fecal contaminated housing and fomites and feed and drinking water can be considered a potential source for infections. In addition to the porcine host, other non-porcine animals on farms may harbor HEV and play a role in environmental persistence (Fig. 4).

#### Manure storages

HEV has been detected in different parts and different types of manure storages. In total six studies report on the presence of HEV in manure pits directly below slatted floors, openings of slurry collecting channels exiting the barn and storages outside the barn, being slurry lagoons, slurry pits and wetlands [49, 75–79]. Pits below slatted floors tested positive on 15 of 22 farms, based on one pooled sample per farm [75]. Slurry from collection channels was positive on 9 of 10 farms, yet in small numbers as only in 2 of 24 samples the virus could be quantified [78].

In all types of storages outside barns, HEV has been detected but none of the studies sampled sufficient farms to statistically analyze associations between HEV presence and a specific storage facility [49, 75–77]. One study does suggest that manure lagoons test positive less often than manure pits due to UV exposure and a different temperature [76]. Another study mentions a higher temperature may enhance biodegradation of organic material including HEV, whereas lower temperatures may preserve virus integrity [75]. Although for HEV it is unknown both how long the virus can survive in swine slurry and what effect manure storage and treatment have on the infectivity of HEV [80], Kasorndorkbua et al. managed to infect pigs with lagoon and pit slurry samples [75]. HEV in slurry may infect pigs through the slatted floors, may contaminate pig feed by fertilizing and irrigating crops, or may contaminate a nearby well that is used for drinking water of pigs. Given the aforementioned findings on HEV in manure storages, we consider manure storage an important environmental source for HEV persistence on farms.

#### Contaminated housing and fomites

Housing or fomites contaminated by another batch of pigs, may be involved in on-farm persistence of HEV. Little is known about survival of HEV in the barn environment or pasture. In one study samples were collected from mobile objects like shovels, panels and fixed objects at height like fans and feed tubes of pig farms and HEV RNA was detected in 3% of the samples inside the farm buildings and 11% of the samples outside the buildings [81]. Unfortunately the manuscript does not mention where inside the buildings HEV was found and how cleaning and disinfection was done on those farms. Other fomites that could hypothetically contribute to HEV persistence are contaminated driving boards, paddles and pen enrichment material. Altogether, knowledge about HEV persistence via contaminated housing is scarce, yet mentioned as important in many studies.

#### Water

Whereas drinking water is a common source of human HEV (gt 1 + 2) infections, for pigs direct proof of this infection source is missing. Only one study investigated HEV presence in water directly from troughs in pig pens and reported one of sixteen tested samples to be HEV RNA positive (6.25%) [50]. Water from hydrants or faucets from 28 farms tested negative in all cases [75]. Unfortunately in these two articles the source of drinking water is not mentioned. In some cases, water taken from streams or wells downstream of pig farm areas have tested positive for HEV but the nearby farms were not tested and another study could not confirm the finding [82, 83]. Many other factors could contribute to the likelihood of finding positive samples of drainage water like the region tested, pig density, type of pig housing (e.g. indoor or outdoor), season et cetera. As water was deemed important in two risk factor studies on pig farms, more research is necessary regarding HEV contamination of drinking water of pigs.

#### Feed

Feed could be a source of HEV to pigs if pigs are fed unpelleted feed. Commercial pelleted pig feed is often sufficiently heated to eliminate thermolabile viruses like HEV. HEV RNA was detected in pig feed products, as was the case in commercial spray dried porcine plasma (SDPP) [84]. Feeding pigs SDPP with HEV RNA however did not result in HEV infections or result in higher level of HEV antibodies compared to a negative control group [84]. Kitchen residues or crops fertilized with pig manure may be contaminated with HEV and attribute to the environmental exposure as source of HEV persistence on pig farms [85].

#### Rodents

The role of rodents in HEV infections on pig farms is interesting, due to their historical association with disease transmission and potential abundance on farms [86]. In rats, HEV-3 RNA has been found, as well as a novel HEV gt, first called ‘rat HEV’ and now known as HEV gt C1 (species *Orthohepevirus* C) [87]. The prevalence of both HEV gt’s in rodents varies from 0 to 18% dependent on the species (mice, *R. rattus* or *R. norvegicus* rats), the location where the rodents are found and the type of samples collected [47, 86, 88–91]. In a study with 63 rats from twelve European countries, all contained HEV gt C1 RNA in their livers [91]. From fifteen states in the USA though, all HEV sequences from the 35 rat livers positive, were related to HEV-3a [90]. Rats and mice trapped around pig farms were positive for HEV-3 in spleens in one study, but are mostly only positive in their intestines [47, 86, 89] or found test negative for HEV-3 [88]. The low prevalence of HEV-3 in rodents around farms and detection of HEV-3 predominantly in intestines, supports the argument that rodents are only accidental hosts of HEV-3. Hence, rodents may not have a significant role in the environmental transmission and persistence of HEV in pig farms. Mechanically, they may contribute to spread of porcine fecal material and as such contribute to environmental contamination, but evidence for this is currently lacking.

#### Probable mechanisms of HEV persistence on pig farms

In summary, important risk factors for HEV transmission and farm persistence include mixing of pigs and improper cleaning and disinfection. Presumable environmental sources of persistence on farms that can spur transmission are manure storages, housing and fomites and water of certain sources.

## Discussion and HEV risk mitigation strategies

The purpose of this review was to summarize and interpret literature about HEV infection dynamics and persistence, to come to a risk mitigation strategy for HEV on farms to ultimately lower the proportion of HEV infected pigs at slaughter.

As far as the authors know, all available English scientific literature on the distinguished topics has been reviewed up to May 2020. We discussed HEV transmission on farms using a compartmental transmission model, to understand the mechanisms of transmission and properly evaluate observational studies and case reports, with regards to study design, low sample sizes and improper study of confounding factors.

We have found that active HEV infections in pigs at slaughter are a consequence of late (re)introduction of HEV in pigs and potentially of environmental on-farm persistence. The infection dynamics differ notably between farms and studies and are influenced by numerous risk factors displayed in Fig. 3.

Furthermore, we have concluded that HEV likely persists on pig farms. Persistence seems to be caused by a constant influx of susceptible pigs, combined with exposure to environmental sources like manure storages or the drinking water well. The environmental compartment En in the mathematical model plays a pivotal role in infection dynamics of pig farms. Still one must consider that finding HEV RNA in the environment – as in any sample – does not warrant viable and intact HEV particles.

Keeping in mind the high farm-level prevalence and probable persistence of HEV on farms, the only strategy for mitigation at the moment is to prevent transmission of HEV between farm compartments. This strategy implies improvement of internal biosecurity. Cleaning and disinfection routines can contribute to increasing the decay rate of HEV in the environment (*η*) according to the mathematical model. Cleaning and disinfection of pens, gates between pens and compartments and fomites that are used in different compartments are prudent. Besides cleaning and disinfection, limiting animal mixing, due to regrouping, would result in less HEV transmission within farms, based on the identified risk factors. On nearly every farm, pigs are regrouped at the start of a new production phase. Based on the fact that particularly pigs early in the fattening stage are shedding, the most risky animal mixing practice would be i) during the transition from nursery to fattening stage and ii) by putting young animals in the same compartment as fattening pigs that have already been there for some weeks (improper all-in-all-out). Improving animal flow measures and cleaning and disinfection will contribute to HEV control.

A future mitigation strategy may be vaccination of pigs, and thereby moving pigs from S to R in the mathematical model. Currently no commercial animal HEV vaccine exists, however two vaccine candidates have shown to confer protection against gt 3 both in animals and humans. The first is a three-dose intramuscular vaccine derived from a gt 4 strain, HEV p179, which has been tested in trials with humans, mice and rabbits and appears to offer (cross) protection against gt 3 and 4 strains [92–94]. The second is a three-dose oral vaccine consisting of proteins derived from a gt 3 strain and immunobiotic bacterium-like particles. The oral vaccine aims to induce an immune response at the site of infection and both a cellular and humoral (IgG and IgA) response have been shown in mice [95], but trials in pigs are not yet reported.

The theoretical effect of vaccination on HEV transmission dynamics in pigs was studied by Backer et al. [96]. They studied three parameters that could be affected by vaccination; mean infectious period, transmission rate and susceptibility. Additionally, the effects of either early vaccination (before weaning) or delayed vaccination (at 10 weeks of age) were studied. Reducing the mean infectious period was effective to decrease the number of infectious pigs at slaughter. Conversely, a lowered rate of transmission or a decrease in susceptibility both flattened the epidemic curve, therefore increasing the probability of infectious pigs at slaughter [96]. Interestingly, the decrease in susceptibility leads to a larger increase of infectious pigs at slaughter with early than with delayed vaccination [96]. Based on the effects of changing the three parameters, Backer et al. concluded that vaccination should either focus on shortening the infectious period, or eliminating the virus from a population, as otherwise, vaccination might lead to an increase in the prevalence of HEV at slaughter. In order to achieve elimination from the population, they demonstrate that a future vaccine must accomplish a reduction factor of around 75% for either of the three parameters in order to eliminate the virus from a herd. Therefore, when an authorized porcine HEV vaccine becomes available, the vaccination coverage and timing of vaccination should be carefully chosen.

In conclusion, based on current knowledge, effective risk mitigation aimed at reducing the proportion of actively HEV-infected pigs at slaughter should be targeted at improving internal biosecurity on farms and in the future possibly at vaccinating pigs. Future research may consider focus on environmental HEV reservoirs in herds and which factors explain the variation of HEV transmission dynamics between farms to reduce the proportion of HEV infected pigs at slaughter.

## Data Availability

Data sharing is not applicable to this article as no datasets were generated or analyzed during the current study.

## Abbreviations

ADV: Aujeszky’s disease virus
CI: Confidence interval
cred. int.: Credibility interval
d.u.: Detectable units
GE: Genome equivalent
Gt: Genotype
HEV: Hepatitis E virus
HEV-1: HEV genotype 1
IV: Intravenous
MSEIR-En: Compartmental model with compartments Maternally derived immunity – Susceptible – Exposed – Infected – Recovered – Environment
NA: Not provided or not studied
PCV2: Porcine circovirus 2
p.e.: Post exposure
p.i.: Post infection
PI_50_: Dose with 50% probability of infecting pig
PRRSV: Porcine Respiratory and Reproductive Syndrome virus
SDPP: Spray dried porcine plasma
